# Advances in the study of the correlation between insulin resistance and infertility

**DOI:** 10.3389/fendo.2024.1288326

**Published:** 2024-01-26

**Authors:** Ruobing Lei, Shuyi Chen, Weihong Li

**Affiliations:** Reproductive Medical Center, The First Affiliated Hospital of Chongqing Medical University, Chongqing, China

**Keywords:** insulin resistance, infertility, assisted reproductive technology, adverse pregnancy outcomes, offspring health

## Abstract

This is a narrative review of the progress of research on the correlation between insulin resistance and infertility. Insulin resistance (IR) is not only involved in the development of various metabolic diseases, but also affects female reproductive function, and to some extent is closely related to female infertility. IR may increase the risk of female infertility by activating oxidative stress, interfering with energy metabolism, affecting oocyte development, embryo quality and endometrial tolerance, affecting hormone secretion and embryo implantation, as well as affecting assisted conception outcomes in infertile populations and reducing the success rate of assisted reproductive technology treatment in infertile populations. In addition, IR is closely associated with spontaneous abortion, gestational diabetes and other adverse pregnancies, and if not corrected in time, may increase the risk of obesity and metabolic diseases in the offspring in the long term. This article provides a review of the relationship between IR and infertility to provide new ideas for the treatment of infertility.

## 1 Introduction

Metabolic Syndrome (MetS) is a group of pathological syndromes characterized by insulin resistance, abdominal obesity, hypertension and hyperlipidemia, and this new non-communicable disease has now become a major health hazard in our society (1). MetS has a strong negative impact on female reproductive function, and metabolic disorders in the body can cause abnormal regulatory functions such as ovarian and other abnormalities in the female ovaries, hormonal imbalances and gonadal dysfunction, which can lead to an increase in infertility incidence (2). Insulin resistance was first proposed by Reaven in 1988 as a central component of this set of abnormalities and plays a role in the development of the disease that cannot be ignored (3). Insulin resistance (IR) refers to a state of hyperinsulinemia caused by a variety of reasons, in which the compensatory secretion of insulin is increased to maintain a stable level of blood glucose in the body due to a decrease in the ability of insulin to take in and utilize glucose (4). IR is involved in the development of many metabolic diseases, including obesity, hypertension, atherosclerosis, polycystic ovary syndrome (PCOS), and nonalcoholic fatty liver disease. As research progresses, the effects of IR on reproductive function are receiving more and more attention, as IR not only increases the chance of infertility in women with PCOS (5), but also increases infertility in non-PCOS women of reproductive age with sporadic menstruation (6). It also increases the risk of recurrent embryo transfer failure during assisted reproductive treatment in infertile women, which can seriously affect the reproductive outcome of patients.

Infertility is an important medical and social problem faced by couples of reproductive age who have not used any contraception and have had regular sex for at least 12 months without obtaining a pregnancy (7). It is an important medical and social problem worldwide. The main causes of female infertility include pelvic tubal factors and ovulation disorders. Among them, endocrine factors such as luteal insufficiency, hyperprolactinemia, polycystic ovary syndrome and thyroid dysfunction can affect egg development, ovulation and endometrial tolerance through multiple stages, increasing the risk of infertility and causing menstrual abnormalities. Obesity can also affect fertility through abnormalities in lipid metabolism and insulin metabolism, and is an independent risk factor for female infertility (8).

The disease that has received the most clinical attention in women with IR is PCOS, but in the infertility population, IR does not occur exclusively in patients with PCOS, but also in non-PCOS patients, more commonly in patients with ovarian polycystic pattern, menstrual sparseness and obesity. Some studies suggest that IR exists independently of PCOS and is a prevalent pathology in infertile women (9).

There have been published reviews on the correlation between IR and infertility in women with PCOS or in women without PCOS, but no review has yet addressed women with PCOS and women without PCOS together. This review not only describes the correlation between IR and infertility in PCOS women and non-PCOS women, but also describes the impact of IR on the outcome of assisted reproduction and pregnancy complications and offspring development.This review reviewed the relationship and controversy about the relationship between IR and endometrial tolerance, summarized the treatment approaches for PCOS combined with IR, and elaborated the related mechanisms and potential therapeutic targets of IR leading to RIF, with the aim of exploring the related mechanisms and providing theoretical basis for further reducing the chances of infertility in patients with IR and improving the reproductive outcomes of IR patients.

## 2 The effect of IR on reproductive function in the infertile population with PCOS

Polycystic ovary syndrome (PCOS) is a common metabolic abnormality combined with reproductive disorders in women of reproductive age, with a prevalence between 5-10% (10), it is a serious threat to women's reproductive health and quality of life. And the clinical phenotype of PCOS is diverse and the diagnostic criteria have not been fully standardized. Currently, several national and professional organizations have issued diagnostic criteria, including the European Society of Reproduction and Embryology (ESHRE), the National Institutes of Health (NIH), the Rotterdam Diagnostic Criteria (11). In 2018, the Chinese Society of Obstetrics and Gynecology also released its own diagnostic criteria (12). Among them, the 2004 Rotterdam criteria are the most widely used diagnostic criteria worldwide, which include sparse ovulation and/or anovulation, clinical and/or biochemical evidence of hyperandrogenism, and ultrasound suggestive of ovarian polycystic changes, two of the above three criteria need to be met, and other factors leading to hyperandrogenemia are excluded to diagnose PCOS (11). The onset of PCOS shows familial aggregation. Studies have shown that the likelihood of the disease occurring within a patient's family is much higher than in other families with five PCOS (13). The environment and poor lifestyle affect human health and also aggravate the metabolic disorders of PCOS. Some toxic and hazardous substances in the environment, especially “environmental endocrine disruptors (EED)”, such as detergents containing nonylphenol and organophosphorus/chlorine pesticides, can enter the human body in a direct or indirect way, affecting human hormone metabolism. Bisphenol-based propane enhances ovarian androgen secretion in most animals. And in PCOS patients due to the accumulation of bisphenol-based propane in the body. It can slow down and reduce the clearance of excess androgens from the body, and it can also cause IR (14). A study (15) demonstrated that female fetuses that are born in a hyperandrogenic uterine environment have a much higher likelihood of exhibiting features of PCOS in adulthood than female fetuses that are born in a relatively normal androgenic environment. Patients with PCOS are at high risk for developing metabolic syndrome (MS), and age and obesity are key factors in determining the prevalence of metabolic syndrome in different populations. Age and obesity are key factors in determining the prevalence of metabolic syndrome in different populations. The prevalence of MS in patients with PCOS has been inconsistently reported due to differences in ethnicity and dietary structure in various regions, as well as the application of different diagnostic criteria for MS. According to a study of the US population (16) data show that the prevalence of MS is about 37% in adolescent PCOS patients and 34. 4% ~ 47. 9% in adult PCOS patients. The results of the study of metabolic syndrome in PCOS patients in Asian countries (17) showed that the prevalence of MS in women with PCOS under the age of 25 years, 25 ~ 30 years, and over the age of 30 years were 22. 5%, 27. 8%, and 53. 5%, respectively. In Brazil, the prevalence of metabolic syndrome in PCOS patients aged 20-24, 25-29, and 30-34 years was 12.1%, 31.7%, and 42.9%, respectively (18). PCOS is a common group of female infertility patients with ovulation disorders, often combined with IR, hyperandrogenemia/clinical manifestations and obesity. An increasing number of studies have shown (19) The interaction between IR, hyperandrogenism and obesity leads to abnormal energy metabolism, impaired follicular development, abnormal oocyte quality, abnormal ovulation, impaired endometrial function and increased risk of thrombosis, resulting in poor embryo quality, non-fertilization and increased risk of miscarriage.

### 2.1 IR aggravates hyperandrogenemia leading to ovulation disorders

Patients with PCOS have clinical or biochemical indicators of hyperandrogenism, with approximately 80-90% of patients having elevated circulating levels of androgens (20). Patients with PCOS have altered gonadotropin secretion, resulting in an increased luteinizing hormone (LH)/follicle stimulating hormone (FSH) ratio. High levels of LH stimulate follicle membrane cells to produce more androstenedione and androgens. On the other hand, the relatively low synthesis and secretion of FSH results in delayed follicular development, decreased aromatase activity in granulosa cells, and insufficient conversion of androstenedione to estrogen, resulting in increased androgens in the ovary (21). Hyperinsulinemia compensates for insulin resistance as a consequence of overstimulation of non-insulin-sensitive tissues, such as the ovaries. In particular, insulin and LH act synergistically on follicular cells to stimulate ovarian androgen production (22). In addition, insulin acts both directly as a gonadotropin, enhancing LH activity by stimulating the expression of LH, insulin, and IGF (insulin-like growth factor 1) receptors on granulosa cells, and indirectly, by impairing the regulation of the hypothalamic-pituitary-ovarian axis. Hyperinsulinemia enhances the adrenal steroid response to ACTH stimulation and reduces the synthesis of sex hormone-binding globulin (SHBG) in the liver, which leads to an increase in total androgen and free androgen levels (23). In addition, hyperinsulinemia inhibits the production of IGF-1-binding protein in the liver.IGF-1 is responsible for triggering androgen production in cells, and inhibition of IGF-1-binding protein production leads to an increase in the concentration of this substance in the circulation, which then produces higher levels of androgens in membrane cells (24). Hyperandrogenemia further aggravates IR, creating a vicious cycle (25).

### 2.2 Association between GLUT4 and impaired endometrial tolerance

Adequate glucose metabolism is essential for the differentiation and maturation of endometrial cells, and glucose transport is mediated by the insulin-mediated glucose transporter factor-4 (GLUT4), which plays an important role in the maturation of endometrial glandular tissue. Fornes et al. (26) showed that the levels of GLUT4 and several proteins of the insulin pathway and their phosphorylation were decreased in the endometrial tissues of PCOS patients, suggesting that there may be local IR in the endometrium of PCOS patients. Low expression of GLUT4 and reduced glucose transport function would cause glucose deficiency in the endometrial cells, which would affect the normal growth of the endometrium and lead to embryo implantation failure or increased risk of miscarriage. Studies have shown that the development and quality of oocytes from IR patients are not affected by *in vitro* culture. Unfortunately, the pregnancy rate was significantly decreased, suggesting that the underlying cause of IR-induced decrease in pregnancy rate is endometrial functional impairment and impaired embryo attachment (27). Tumor necrosis factor-α (TNF-α) is expressed at higher levels in the skin adipose tissue of women with PCOS than in the general population. In human endometrial stromal cells, excess TNF -α negatively affects insulin sensitivity and leads to abnormal energy metabolism by decreasing lipocalin signaling and blocking GLUT-4 membrane transport (28), which may be one of the reasons for the impaired endometrial receptivity in patients with PCOS. In addition, it has been found that reduced cortisol oxidation may inhibit the insulin signaling pathway by inducing PTEN expression in endometrial epithelial cells of women with PCOS, thereby promoting endometrial IR (27). However, the study by Lee et al. tentatively failed to detect GLUT4 mRNA in the endometrium of PCOS or controls (29). Thus, the relationship between PCOS-associated IR and impaired endometrial tolerance remains controversial and requires further study.

### 2.3 Oxidative stress and inflammatory factors exacerbate IR

Oxidative stress (OS) is an imbalance between the body’s oxidative and antioxidant systems under normal physiological conditions, and the body produces excess reactive oxygen species (ROS) under oxidative stress, which can cause oxidative damage to the body, resulting in apoptosis, tissue damage, and a variety of diseases. Recent investigations have shown that most PCOS patients are in a state of oxidative stress, and the incidence of oxidative stress is highest in patients with combined IR and infertility (30). It was found that (31) IR-induced ROS formation disrupts the function of mitochondria and subsequently affects the regulation of mitochondrial oxidative phosphorylation. After mitochondrial damage, the generated ROS can activate the inflammatory factor TNF-α, which has a detrimental effect on insulin signaling, and also, TNF-α can interact with interleukin 1β (IL-β) and interleukin 6 (IL-6) to make the function of pancreatic β cells impairs the function of pancreatic β-cells, which in turn aggravates IR (32). ROS directly or indirectly activate the JNK/SAPK pathway and promote serine/threonine phosphorylation of insulin receptor substrate (IRS), which further induces IR (4).

### 2.4 Multiple treatments for insulin resistance in polycystic ovary syndrome and their effectiveness

The findings of Marcondes et al. (33) showed that the prevalence of MS in normal weight, overweight, obese, and severely obese PCOS patients were 0, 23. 8%, 62. 9% and 85. 5%. Weight reduction through lifestyle intervention remains the first-line treatment for overweight or obese women with PCOS. In terms of diet, patients should be encouraged to reduce the intake of saturated fats and refined carbohydrates and increase the intake of protein-rich foods, which can increase the patients’ satiety and improve insulin sensitivity (34). Currently, in clinical practice, the main therapeutic goal is to reduce patients’ symptoms by regulating their hormone and plasma insulin levels. Metformin can be used to intervene in PCOS patients with impaired glucose tolerance. Metformin can improve the abnormal metabolism of insulin levels and regulate blood glucose concentration to maintain it in the normal range and improve IR, and it can also regulate the menstrual cycle so that the ovaries can restore normal ovulation function (35). In view of the complexity of the etiology of PCOS, it is difficult to achieve significant therapeutic effects with a single drug, so several drugs are mostly used in clinical practice for combination therapy, and certain efficacy has been achieved.

## 3 Effect of IR on reproductive function in a non-PCOS infertile population

In fact, not all infertile women have PCOS, and IR does not occur only in those with PCOS. With changing lifestyles, more and more women are actively or passively smoking and consuming large quantities of beverages such as coffee, cocoa, tea, chocolate and cola-type beverages, which are not conducive to women’s reproductive health (36). The menstrual cycle is a basic biorhythmic cycle that occurs in women of childbearing age and is controlled by rhythmic fluctuations of hormones within the hypothalamic-pituitary-gonadal axis, whereas the uterine cycle consists of a menstrual phase, a proliferative phase, and a secretory phase, which is aimed at preparing a favorable endometrial environment for embryo implantation, and disruption of the menstrual cycle will also contribute to the development of infertility in women (37). Many studies have shown that menstrual cycle dysfunction is associated with insulin resistance (38) and that women with long menstrual cycles (36-45 days) have more severe endocrine abnormalities, and a higher risk of spontaneous abortion, infertility and anovulation (6). In the context of the natural menstrual cycle, small but significant changes in insulin and insulin resistance index (HOMA-IR) correspond in part to the levels of estradiol and progesterone. There has been report that during the menstrual cycle, HOMA-IR increased from a level of 1.35 in the mid-follicular phase to a level of 1.59 in the early luteal phase, and then decreased to 1.55 in the late luteal phase, with positive correlations with estradiol and progesterone and negative correlations with FSH and SHBG (39). In a study on the effect of insulin resistance on the outcome of *in vitro* fertilization in non-PCOS body lean women, it was found (40) the number of oocytes and the risk of developing exogenous gonadotropin-stimulated multiple follicle production or ovarian hyperstimulation syndrome were higher in the IR group than in the non-IR group, and the percentage of matured oocytes and the percentage of freezable blastocysts per residual embryo were significantly lower in IR than in the women in the non-IR group, suggesting that IR also plays an important role in infertility in non-PCOS women. women with IR are more likely to be overweight or obese, factors that can also affect reproductive function. It is believed that there is an “adipose-insulin axis” in the body, and leptin plays a negative feedback role between fat and insulin. Due to leptin resistance in obese patients, the inhibitory effect of leptin on insulin secretion is weakened, which interferes with the “adipose-insulin axis”, and thus also aggravates hyperinsulinemia. This also aggravates hyperinsulinemia or insulin resistance. In addition, hyperinsulinemia and excess adipose tissue release large amounts of long-chain saturated fatty acids, leading to increased oxidation, resulting in anaerobic oxidative capacity of follicular cell mitochondrial and endoplasmic reticulum dysfunction, granulosa cell apoptosis, hormone synthesis, and damage to the ovarian localization and the follicular development of the microenvironment (41). Abnormal free fatty acid (FFA) levels in female follicular fluid affect oocyte maturation and development, which in turn affects oocyte growth and differentiation (42). Obesity can cause elevated levels of FFA in follicular fluid, insulin can inhibit the release of FFA from adipose tissue, and the presence of IR can attenuate this effect, leading to the accumulation of FFA in the blood. Studies have shown that (42) in follicular fluid, the types of FFA are consistent with those in plasma and correlate with the levels in plasma, suggesting that in the IR state, the concentration of FFA in follicular fluid increases, further affecting the growth and development of follicles in IR patients. Lifestyle modification is the most important therapeutic measure in infertility due to IR, followed by pharmacologic therapy. Since metformin often causes side effects, new integrative strategies have been proposed to treat insulin resistance, such as the use of inositol. Myo-inositol (MYO) and d-chiral inositol (DCI) are the two stereoisomers of inositol in humans. MYO is the precursor of inositol triphosphate, the second messenger for the regulation of thyroid-stimulating hormones (TSH) and FSH, as well as insulin. Several preliminary studies have suggested that deficiency of DCI containing inositol phosphor glycans (IPG) may underlie insulin resistance (43). Indeed, Genazzani et al. reported that MYO administration not only reduced fasting insulin plasma levels in obese patients, but also increased insulin sensitivity in non-obese PCOS patients (44, 45). One study (46) found no significant differences in clinical pregnancy rates, live birth rates, and miscarriage rates between women with hyperinsulinemia (HI) and IR compared to those without PCOS. Another study found (47) that most insulin-resistant women exhibit single follicle development and that treatment of infertility in anovulatory women with clomiphene citrate (CC) improves their clinical pregnancy rates; therefore, insulin resistance can be considered an important factor contributing to anovulation. Further studies are needed on the effect of IR on pregnancy outcomes and treatment in non-PCOS women.

## 4 IR affects assisted reproduction pregnancy outcomes in infertile patients

Adult women under 40 years of age who have failed to achieve clinical pregnancy after transfer of at least 3 quality embryos in 3 fresh or frozen cycles, where quality embryos include: day 3 embryos (≥8 cells, uniform ovoid bulb size, fragmentation rate <10%) and blastocysts (≥3BB), are considered as repeated implantation failure (RIF) (48). RIF occurs in 15-20% of infertile couples undergoing IVF-embryo transfer (ET) for pregnancy (49). Several studies have shown that IR can affect the outcome of assisted reproductive treatment (ART) in infertile patients by affecting oocyte growth and development, embryo quality and endometrial tolerance.

### 4.1 IR affects oocyte quality

Oocyte quality in general tends to decline as women age. Age affects oocyte and embryonic developmental potential by affecting the granulosa cells and follicular fluid and altering the microenvironment outside the oocyte (50). In older women, there is a decrease in antioxidant capacity, a decrease in free radicals, and a buildup of reactive oxygen species (ROS), which cause damage to cellular structures and lead to stagnation of follicular development (51). High quality mature oocytes and embryos are the key to ART success. High quality oocytes are more likely to be fertilized to produce quality embryos and have higher fertilization and cleavage rates, which can enhance clinical pregnancy rates and improve patient prognosis (52). Therefore, a decrease in the number and quality of oocytes has a significant impact on the subsequent development and implantation of embryos.

#### 4.1.1 IR damages oocytes via telomeres and spindle

A telomere is a complex of specialized repetitive DNA sequences and sheltering proteins located at the ends of eukaryotic chromosomes. Its basic function is to protect linear chromosome ends from being mistaken for a broken end being incorrectly repaired by the cell itself or undergoing processes such as DNA end joining or DNA recombination (53). Telomere length is critical for maintaining the divisional capacity of oocytes, and once telomeres are depleted, the cell will immediately initiate the apoptotic program and progress toward apoptosis (54). One mechanism by which IR-induced hyperglycemic states may promote telomere shortening is through oxidative stress, which produces oxygen free radicals that affect telomere ends, which are enriched with guanine residues, the latter of which make them susceptible to oxidative stress (55). Oocytes are prone to chromosome segregation errors during meiosis, resulting in aneuploidy, and these factors include crossover problems when recombination occurs in meiotic M I, spindle assembly checkpoint (SAC) deletion, and so on, which may independently induce aneuploidy in oocytes, or they may be interrelated (56). IR has the same effect on the normal function of the spindle in the nucleus, resulting in deletion of SAC protein and paradoxical segregation pull of the spindle on the chromosomes, leading to aneuploidy or polyploidy, which further affects the chromosomes of the oocyte and the post-fertilized embryo, leading to failure of embryo implantation or miscarriage (57, 58).

#### 4.1.2 IR affects oocytes through oxidative stress and impairment of mitochondrial function

Mitochondria are the main productive organelles in the oocyte cytoplasm, the main producers of ROS, and the first productive organisms exposed to oxidative stress, and are closely related to oocyte quality.IR can cause increased ROS production during oocyte maturation, causing an oxidative/antioxidative imbalance in the oocyte cytoplasm and reducing mitochondrial enzyme activity and its antioxidant capacity, which in turn impairs mitochondrial function (59). As an exposed circular double-stranded structure distributed in the mitochondrial matrix and endosomal membrane, mtDNA lacks protection from histones and is constantly generating ROS from the electron transport chain in the mitochondrial endosomal membrane, and long-term exposure to high oxidizing environments makes it more susceptible to mutation and mitochondrial dysfunction than nuclear DNA (60). Many of the mutations in mtDNA are undetectable, usually only detectable when a woman enters her reproductive years, and the chances of a successful pregnancy are greatly reduced after a certain level of mutation. Mitochondria need to undergo fusion and fission to maintain their highly dynamic and changing structure. Mitochondrial fusion protein 1 (MFNl) and MFN2 mediate mitochondrial fusion and its interaction with the endoplasmic reticulum (61). Experiments have shown (61) that the absence of MFN1 in oocytes down-regulates calreticulin and connexin, leading to follicular arrest at the secondary follicular stage, impaired communication between oocytes and granulosa cells, increased accumulation of ceramides, which in turn induce apoptosis of oocytes, and the absence of its targeting also accelerates follicular depletion, ultimately leading to infertility in women. Oocyte-specific deletion of MFN2 also causes impairment of mitochondrial function and dynamics, which further leads to impaired follicular development and oocyte maturation, telomere shortening, and an increase in apoptosis, which in turn leads to a series of problems such as reduced oocyte quality and follicle depletion (62).

### 4.2 IR causes embryonic developmental arrest

Studies have shown that (63) mammalian target of rapamycin (mTOR) is an important regulator of embryonic developmental arrest, and culturing embryos in the presence of mTOR can arrest embryonic development at the blastocyst stage for 9-12 days (64). mTOR plays an important role in nutrient supply and metabolism *in vivo* and is associated with multiple signaling pathways *in vivo*, acting in concert with its upstream and downstream signaling molecules to affect cell proliferation and apoptosis (65). IR can activate mTOR through oxidative stress (32). Therefore, in the maternal state with IR, mTOR signaling is compromised, leading to early embryonic developmental arrest.

### 4.3 IR affects endometrial tolerance

The development of infertility is closely related to abnormalities in the embryo implantation process, and the tolerability of the endometrium is crucial for successful embryo implantation. During embryo implantation, the endometrium is in a state that allows embryo attachment and invasion, causing stromal changes in the endometrial tissue, and ultimately, successful embryo implantation. IR can impair endometrial receptivity by inhibiting decidual differentiation. Close coordination of the decidua is essential for the process of embryo implantation and development. Its main functions are to protect the maternal-fetal interface from oxidative stress, to suppress certain maternal immune responses, and to control the migration and invasion of extrachorionic trophoblast cells (66). IR-induced hyperandrogenemia regulates endometrial cell growth and activity by interfering with glucose metabolism, thereby inhibiting decidual differentiation and preventing embryo implantation (67). Studies have shown that (68), IR can put the endometrium in a state of insufficient energy supply and increased demand, while activating the AMPK signaling pathway, which further affects intracellular glucose metabolism. However, further basic experiments are needed to investigate the underlying mechanisms by which the AMPK signaling pathway affects endometrial tolerance. High levels of insulin *in vivo* regulate apoptosis through the PI3K-Akt pathway, significantly reduce the number of receptors for bovine serum albumin 2 (BMP2), a decidual marker, estrogen and progesterone, further damage endometrial stromal cells, and increase the mitochondrial transmembrane potential, affecting endometrial decidualization (69).

### 4.4 IR-induced pathway damage and effectiveness of pathway therapy

Recent animal experiments and clinical trials have shown that antioxidants such as resveratrol, coenzyme Q10, melatonin, folic acid, and vitamin E may improve the quality of human oocytes and embryos by improving mitochondrial function and promoting mitochondrial biosynthesis, and improve the fertility outcomes of infertile patients with PCOS and early onset ovarian insufficiency. Oral antioxidants prior to the cycle or the addition of antioxidants to *in vitro* immature oocyte cultures in patients with assisted reproductive fertilization may improve fertility outcomes (70). Progress has also been made regarding targeted therapies for impaired insulin signaling pathways. The major signaling regulatory pathways of IR include insulinreceptorsubstrate1 (IRS1)/phosphatidylinositol-3-kinase (PI3K)/serine- serine-threoninekinase (Akt) pathway, mitogen-activated proteinkinase (AMPK) pathway, Smad3 pathway, and so on. Recent studies have found that the receptor for advanced glycation end products (RAGE) can reduce insulin sensitivity in tissues critical for glucose metabolism through mediated downregulation of the AMPK downregulation of the Akt signaling pathway, Silencing the signals transmitted by this receptor may be a therapeutic strategy, and the use of RAGE antagonists could potentially improve insulin receptor signaling and enhance insulin sensitivity in patients with impaired glucose tolerance (71). LKB1 is a serine/threonine kinase, which acts as an upstream kinase of AMPK and can directly phosphorylate AMPK involved in glycolipid metabolism and regulate the onset and progression of IR (72). Ma et al (73) established a rat model of PCOS and IR and found that ubiquitin protease E3A (UBE3A) mRNA levels were significantly increased, and UBE3A mRNA levels were further increased in PCOS combined with IR rats. Knockdown of UBE3A decreased ubiquitination of AMPK and enhanced AMPK phosphorylation, and the LKB1/AMPK signaling pathway was activated. p-IRS1 and p-AKT levels were significantly downregulated in PCOS combined IR rats after UBE3A knockdown after AMPK knockdown, suggesting that silencing of UBE3A inhibits the progression of IR in PCOS through the AMPK pathway. UBE3A may be a potential biological target for clinical treatment of IR and PCOS in the future.

## 5 IR and pregnancy complications

During pregnancy, IR is a physiological change that ensures normal fetal growth and development and maintenance of normal maternal blood glucose levels, but excessive or pathological IR is an important factor leading to complications during pregnancy.

### 5.1 IR and recurrent miscarriage

Recurrent spontaneous abortion (RSA) refers to 2 or more pregnancy losses with the same partner, including biochemical pregnancies, before 28 weeks of pregnancy (74). IR can contribute to miscarriage through mechanisms such as inflammation, immunity, energy metabolism, and the induction of a prothrombotic state.

#### 5.1.1 IR leads to RSA through inflammatory response

IR plays an important role in the development of the hyperglycemic state in patients, and hyperglycemia activates the inflammatory response, which over time leads to a long-term chronic inflammatory state in the body, ultimately leading to increased islet damage IR.Glucose is a major product of the redox process in mononuclear circulating cells and when oxidized, it leads to the formation of ROS, which activates the NF-κB inflammatory pathway and increases the levels of inflammatory factors such as TNF-α, IL I6 and CRP (75). Inflammatory factors also stimulate and generate large amounts of ROS, and in the human body, pancreatic β-cells are more susceptible to free radical attack and oxidative damage than other cells within body tissues. In addition, due to the high expression of oxidative stress products (OS) in the body, the DNA content in islet β-cells can decrease, which in turn causes a decrease in the number of β-cells in the islets, deteriorating their function and thus exacerbating IR. Chronic inflammation due to increased inflammatory factors increases the expression of cyclooxygenase-2 (COX-2), which in turn increases the expression of vascular endothelial growth factor, ultimately affecting endometrial vascular permeability and angiogenesis of the endometrium (76), which can lead to fetal loss due to impaired maternal-fetal blood flow supply.

#### 5.1.2 IR triggers RSA through immunization

Antiphospholipid antibodies (APLs) refer to autoimmune antibodies that act on phospholipids and/or phospholipid-binding proteins and include mainly anticardiolipin (ACL), 1upus anticoagulant (LA) and anti-β2-glycoprotein I (β2GP1) antibodies. LA acts in the body against its target antigens and is resistant to natural anticoagulant pathways, and can also promote coagulation by inhibiting fibrinolysis, damaging endothelial cells, and activating platelets, leaving the pregnant person in an abnormally hypercoagulable state, leading to adverse pregnancy outcomes such as thrombosis and miscarriage (77, 78). In terms of the risk of thrombosis caused by APLs, the rate of LA positivity is higher than that of β2GPl-Ab or ACL positivity (79). In contrast to ACL and β2GP1-Ab, LA is an important factor in the occurrence of adverse pregnancy outcomes (80). Zhang Chunfang et al. (81) showed that LA levels were significantly increased in RSA patients with concomitant IR compared to RSA patients without IR, and LA levels were positively correlated with HOMR-IR, fasting insulin values (FINS), 2-hour glucose after glucose intake (2h-PG) and 2-hour insulin (2h-INS), suggesting that IR may contribute to RSA by inducing the production of LA and/or exacerbating its abnormalities,leading to RSA. In one study (82), in unexplained early abortion, the T cell-mediated immune response is found hyper-activated, leading to hyper-immunosuppression at the maternal-fetal interface and maternal rejection of the fetus and its appendages. This immune response not only limits the nutritional supply to the embryo, but may also cause embryonic damage, leading to embryonic hypoplasia or embryonic arrest, resulting in RSA (83).

#### 5.1.3 IR induced prothrombotic state leading to RSA

The prothrombotic state is strongly associated with RSA, and a significant hypercoagulable state may increase thrombotic tendency and make the placenta more susceptible to microthrombosis, which may adversely affect the fetal blood supply and cause adverse pregnancy events such as miscarriage. The mechanism of hyperinsulinemia-induced prothrombotic state formation may be related to the triggering of elevated plasma levels of homocysteine (Hcy) and fibrinogen activator inhibitor 1 (PAI-1) (84, 85). Studies (86) Hcy can affect the normal growth cycle of embryonic cells by influencing DNA methylation, which can lead to apoptosis. Various studies have shown that Hcy can accelerate cellular senescence through multiple mechanisms. In a study by Zhang et al. (87), endothelial cells cultured in an Hcy environment could lead to cellular senescence or apoptosis through DNA hypomethylation by human telomerase reverse transcriptase (hTERT), which resulted in an increase in markers of cellular senescence acidic β-galactosidase. In addition, Hcy upregulated markers of cellular senescence, including p16, p21, and p53, in cultured endothelial cells; however, administration of folic acid or S-adenosylmethionine (SAM) reversed these effects. Redox state imbalance and oxidative stress are considered to be the main mechanisms of pathogenesis associated with HHcy. Meanwhile, Hcy undergoes oxidative reactions *in vivo* to generate ROS and activate epithelial sodium channels, which induce endothelial dysfunction through reactive oxygen species (ROS)/COX-2-dependent activation of SGK-1/Nedd4-2 (serum/glucocorticoid-regulated kinase 1/neural precursor cell expression of developmentally down-regulated protein 4-2) signaling (88), and cause NO production and release is inhibited, leading to increased cellular damage effects such as lipid peroxidation and ultimately endometrial vascularization. The damaged vascular endothelium, in turn, contributes to platelet activation and aggregation, which disturbs the intrauterine environment and leads to early miscarriage (89, 90). The secretion of PAI-1 is stimulated by insulin and may be increased by the induction of Hhcy (91). It inhibits fibrinogen production during fibrinogen activation, causing destruction of vascular endothelial cells by abnormal vascular endothelial factors, resulting in a state of low local fibrinolysis and leading to the formation of a prethrombotic state (92) On the other hand, PAI-1 reduces blood supply during embryonic development, leading to poor trophectoderm development, and on the other hand, it damages the placenta and impairs embryonic development, eventually leading to miscarriage.

### 5.2 IR and gestational diabetes

Gestational diabetes mellitus (GDM) is a complication of pregnancy caused by impaired glucose metabolism (93). GDM prevalence was lowest among non-Hispanic white women (4.2%) and black person (4.4%); it also varied among Asian subgroups: Japanese (5.5%), Koreans (6.7%), Pacific Islanders (7.2%), and Chinese (7.9%), and among Southeast Asians (8.8%), Filipinos (9.6%), and Asian Indians (11.1%) highest (94). Physiologic indices of pregnant women change at each stage of pregnancy, and changes in insulin-related indices during pregnancy may be an important factor in the development of GDM (94). In the middle and late stages of pregnancy, anti-insulin-like substances such as progesterone and estrogen are elevated in the pregnant women’s bodies, which affects the sensitivity to insulin and increases the amount of insulin secreted, which is insufficient to compensate for the combined insulin defects, thus leading to the emergence of GDM (95). Lu Beiyi et al. (96) found that IR and islet beta function in both GDM patients and normal pregnant women enhanced with increasing gestational weeks, but IR levels were significantly higher in GDM patients than in normal pregnant women, and insulin secretory function was lower in GDM patients than in normal pregnant women in early pregnancy, and it was hypothesized that compensatory insulin secretion defect and IR in GDM patients may be an important cause of GDM occurrence. The underlying mechanism seems to be that IR-induced oxidative stress leads to a decrease in the number of pancreatic β cells, which in turn reduces their function and exacerbates IR (76), and when islet β-cell function is not compensated, IR progresses to GDM.

### 5.3 IR and hypertension in pregnancy

Hypertensive disorder of pregnancy (HDP) is a serious perinatal disorder that occurs when pregnancy is accompanied by elevated blood pressure, including gestational hypertension, pre-eclampsia and eclampsia (97). The study was carried out in the United States. Meng Xiyan et al. (98) have shown that IR has an effect on HDP. The mechanism of IR-induced hypertension may be due to a decrease in NO synthesis and disruption of lipid metabolism caused by IR, resulting in endothelial cell damage, which increases peripheral vascular resistance and increases blood pressure (99). IR can also increase the concentration of inflammatory factors such as TNF-α and promote apoptosis of trophoblast cells by macrophages, resulting in superficial placentation, ischemia and hypoxia, which in turn cause endothelial cell dysfunction and systemic small artery spasm, thus triggering the development of HDP (100).

## 6 IR and neonatal outcome

Severe maternal IR, persistent hyperglycemic and hyperinsulinemic states increase fetal weight and significantly increase the incidence of macrosomia. Studies have shown that patients with GDM are more likely to deliver giant babies, mainly due to the state of maternal hyperglycemia caused by IR. Glucose is transported from the mother to the fetus in a labile diffusion manner, and when maternal blood glucose is elevated, fetal blood glucose is also elevated, which in turn stimulates the development of hyperinsulinemia in the fetus, which further promotes the massive synthesis of fetal lipids and proteins, as well as the inhibition of lipolysis, ultimately leading to the formation of a macrosomic child (101). The prevalence of macrosomia varies globally, which may be due to racial differences between populations as well as differences in socioeconomic status. Variations in the effects of GDM and weight characteristics on racial macrosomia may be due to metabolic differences as reflected in changes in body composition and/or genetics. Silva et al. (102) compared the different races of the Hawaiian population and found that, compared to Japanese, Chinese, and white women, Native Hawaiian and Filipino women with GDM were at greater risk of delivering large macrosomic babies, white mothers had the highest rate of macrosomic babies compared to black or Asian mothers, and Latina mothers had a higher incidence of LGA (larger-than-gestational-age) babies (103). Over the past three decades, the number of macrosomic infants has increased from 5% ~ 20% to 15% ~ 25% in developed countries (104) Mothers’ history of macrosomia deliveries and gestational weeks were significantly associated with macrosomia, male children were significantly more likely to have macrosomia than female children, and gestational weeks of 37 weeks and above were also significantly associated with macrosomia (105). A cross-sectional study in China showed that the risk of macrosomia in pregnant women increased with the age of the mother, with a major peak in the incidence of macrosomia at the age of 33 years, and the risk of macrosomia in pregnant women aged ≥35 years increased with age compared with pregnant women aged <35 years (106, 107). A study (108) found that the offspring of mothers with GDM had significantly higher fasting glucose, systolic and diastolic blood pressure, TC, TG and LDL-C than the offspring of mothers with normal values of all indicators. This suggests that GDM due to severe IR increases the risk of diabetes and hypertension in offspring. The American Diabetes Association suggests that pregnant women with poor glycemic control can be guided during pregnancy through medical nutritional interventions as well as lifestyle modifications, which can improve glycemic control and reduce the chance of macrosomia. Therefore, clinically diagnosed GDM patients should be intervened as early as possible, and nutritional control should be based on the patient’s weight and blood glucose level. The purpose of nutritional intervention is to improve the overall nutritional status of the fetus and the mother, to maintain the weight gain during pregnancy within a reasonable range and to reduce the incidence of macrosomia through reasonable energy intake.

## 7 IR and postpartum outcomes

It has been found that insulin resistance in pregnant women with GDM does not return to normal quickly after delivery, and some patients have persistent resistance and even develop type 2 diabetes mellitus (109). Another study found (110) that the prevalence of diabetes mellitus in GDM pregnant women gradually increased at 2, 6, 12, 18 and 24 months postpartum. This suggests that the incidence of abnormal glucose metabolism in pregnant women with GDM is high and gradually increasing at 2 years postpartum. Shen et al. (111) observed that Chinese women with GDM had the greatest risk of developing T2D in the past 10 years, while African American and Caucasian women had a moderate and low risk, respectively. In contrast, in a recent meta-analysis including more than 90,000 women with prior GDM, the highest prevalence of T2D after GDM was associated with black race (112). Domestic and international guidelines for diabetes prevention and treatment emphasize strict control of blood glucose levels during pregnancy in women with GDM, the need for OGTT screening for postpartum glycemic abnormalities at 4-12 weeks postpartum, and lifelong screening for diabetes in order to prevent the development of postpartum diabetes (113). Huvinen et al. (114) showed that women with GDM had elevated levels of TC, TG, and LDL-c in the postpartum period from 22 to 28 years and HDL-c levels decreased and cardiovascular events increased in women with GDM. This suggests that not only the incidence of postpartum diabetes mellitus but also dyslipidemia is very common in GDM patients. However, research on the management and harm of dyslipidemia in GDM is quite limited, and there is no unified diagnostic standard for dyslipidemia during pregnancy and no standardized guideline for the management of dyslipidemia during pregnancy and postnatal period, so dyslipidemia during pregnancy should be actively managed in the clinic, which is conducive to the reduction of the incidence of cardiovascular disease in the postnatal period.

## 8 Conclusion

In conclusion, IR may have a significant impact on the reproductive function of women of childbearing age through multiple mechanisms and is closely related to female infertility. IR increases the risk of infertility, adverse pregnancy outcomes and metabolic abnormalities in the offspring. Therefore, clinical attention and guidance should be focused on the women of childbearing age with IR, and healthy lifestyle and dietary structure modification before pregnancy should be carried out to correct abnormal metabolic indicators in order to improve reproductive outcomes. As the number of factors associated with pathophysiology increases, more and more data suggest that IR is an important contributor to female infertility, and that many factors, including PCOS, obesity, inflammation, and oxidative stress may exacerbate this condition; however, the role of these factors in the pathogenesis of female infertility due to IR is not fully understood. The future effects of IR on oocyte quality, embryo quality, and endometrial tolerance in female females remain to be further explored in depth, and high-quality randomized controlled clinical trials are needed to verify the effects of various interventions and pharmacological treatments on improving the fertility and pregnancy outcomes of women with IR and develop appropriate guidelines for the management of metabolic abnormalities during pregnancy and postpartum in IR women.

## Author contributions

RL: Writing – original draft. SC: Methodology, Writing – review & editing. WL: Writing – review & editing.
